# Controlled Morphological Growth and Photonic Lasing in Cesium Lead Bromide Microcrystals

**DOI:** 10.3390/nano14151248

**Published:** 2024-07-25

**Authors:** Mamoon Ur Rashid, Zeeshan Tahir, Muhammad Sheeraz, Farman Ullah, Yun Chang Park, Faisal Maqbool, Yong Soo Kim

**Affiliations:** 1 Department of Semiconductor Physics & Engineering and Energy Harvest-Storage Research Center, University of Ulsan, Ulsan 44610, Republic of Korea; quantum1@mail.ulsan.ac.kr (M.U.R.); 20175235@ulsan.ac.kr (Z.T.); msheeraz@ulsan.ac.kr (M.S.); faisal1991@mail.ulsan.ac.kr (F.M.); 2 Department of Mechanical & Mechatronics Engineering and Waterloo Institute for Nanotechnology, University of Waterloo, Waterloo, ON N2L 3G1, Canada; fullah@uwaterloo.ca (F.U.); 3 Measurement and Analysis Division, National Nanofab Center, Daejeon 34141, Republic of Korea; parkyc@nnfc.re.kr

**Keywords:** lead halide perovskite, morphology control, growth dynamics, precursor flux, temperature, epitaxial growth, lasing

## Abstract

Morphology plays a crucial role in defining the optical, electronic, and mechanical properties of halide perovskite microcrystals. Therefore, developing strategies that offer precise control over crystal morphology during the growth process is highly desirable. This work presents a simple scheme to simultaneously grow distinct geometries of cesium lead bromide (CsPbBr_3_) microcrystals, including microrods (MR), microplates (MP), and microspheres (MS), in a single chemical vapor deposition (CVD) experiment. By strategically adjusting precursor evaporation temperatures, flux density, and the substrate temperature, we surpass previous techniques by achieving simultaneous yet selective growth of multiple CsPbBr_3_ geometries at distinct positions on the same substrate. This fine growth control is attributed to the synergistic variation in fluid flow dynamics, precursor substrate distance, and temperature across the substrate, offering regions suitable for the growth of different morphologies. Pertinently, perovskite MR are grown at the top, while MP and MS are observed at the center and bottom regions of the substrate, respectively. Structural analysis reveals high crystallinity and an orthorhombic phase of the as-grown perovskite microcrystals, while persistent photonic lasing manifests their nonlinear optical characteristics, underpinning their potential application for next-generation photonic and optoelectronic devices.

## 1. Introduction

Halide perovskites, particularly CsPbBr_3_, have attained significant attention over the past decade owing to their exceptional characteristics, including high quantum efficiency [1,2,3], high carrier mobility [4,5], long carrier diffusion lengths [6,7], large exciton binding energy [3], and low trap densities [8,9]. Unlike organic–inorganic halide perovskite, the absence of hydrophilic organic groups in CsPbBr_3_ ensures its chemical stability, addressing the growing concerns over the longevity and the applicability of halide perovskites for potential device applications [10], such as photovoltaics, solar cells, light-emitting diodes, photodetectors, and lasers [11,12,13,14,15,16]. Therefore, various synthesis routes have been explored to achieve high-quality single crystals in the quest to gain control over morphology and uniformity, which are imperative for the realization of self-hybridized miniature lasers [17,18,19]. This has led to the development of various solution-based synthesis techniques, such as solvothermal, Bridgman, inverse temperature crystallization, and antisolvent precipitation methods. These approaches offer simplicity and scalability but usually suffer from the poor crystalline quality, uniformity, and lack of control over the crystal morphology [17,20,21]. In contrast, vacuum-based deposition methods offer meticulous control over the growth parameters, yielding high-quality perovskite crystals [22,23]. In particular, the CVD technique avoids the use of solvents, thus preventing the formation of grain boundaries and surface defects, yielding high-quality single crystals with controlled morphology and uniformity, making it one of the most widely studied techniques. Moreover, the ability to achieve various morphologies further broadens the spectrum of their applications [24,25,26,27]. 

To date, tremendous efforts have been made to realize perovskite microcrystals with various shapes, including sheets [28,29], microrods [30,31,32], microspheres [33], and pyramids [34], via controlling a multitude of essential parameters, such as precursor–molar ratio, nucleation temperature, gas flow rate, cooling rates, and substrate-to-precursor distance, to name a few [22,23,24,25,33,35,36]. For instance, Shangui et al. reported the growth of plate, pyramid, and sphere-shaped perovskite microcrystals by tuning the evaporation temperature and cooling rates [35], while Xindi. et al. realized microwire, microplate, and pyramid geometries by manipulating the substrate-to-precursor distance inside the CVD furnace [25]. However, it is worth mentioning that the majority of these results involve performing an individual set of experiments for the growth of each geometry, which is not only costly but is also a time-consuming process. Therefore, the quest to simultaneously grow multiple perovskite geometries at selective regions of the substrate in a single experiment persists.

Herein, we demonstrate a facile growth process capable of simultaneously realizing multiple geometries of CsPbBr_3_ (MS, MP and MR) at selective regions of the substrate in a single CVD experiment. For this purpose, a two-zone CVD furnace is used wherein the precursors (CsBr and PbBr_2_) contained in graphite crucibles are placed separately in their respective temperature zones for effective evaporation. A monolithic mica substrate is aligned vertically next to the PbBr_2_ crucible for deposition. Intriguingly, this unique configuration offers a variable precursor flux density and temperature across the selective regions of the substrate, making it ideal for the growth of different morphologies. Accordingly, MR and MS are grown at the top and bottom regions, while MP are obtained at the center of the substrate. Note that the variation in the precursor flux and temperature originates from the cumulative effect of precursor-to-substrate distance, crucible-induced variational fluid flow, and the placement of the heating unit inside the furnace. Moreover, the selective growth of these perovskite geometries is further validated by performing three individual experiments, wherein mica substrates are placed horizontally at three discrete vertical positions corresponding to the top, center, and bottom regions of the vertically aligned mica substrate. Interestingly, a unique geometry is observed at each designated position, confirming the selective growth. Furthermore, the crystalline quality and phase of the as-grown microcrystals are examined via X-ray diffraction (XRD), high resolution transmission electron microscopy (HRTEM), and selected area electron diffraction (SAED), which not only confirm the high crystallinity and orthorhombic phase of the microcrystals but also demonstrate the epitaxial growth of MR and MP geometries. Finally, the nonlinear optical characteristics of the perovskite microcrystals are investigated by demonstrating low threshold photon lasing actions, highlighting their potential applicability for future optoelectronic devices. 

## 2. Materials and Methods

### 2.1. Materials

The target materials CsBr (99.9%), and PbBr_2_ (99.9%) were purchased from Sigma Aldrich (Seoul, Republic of Korea) while MICA Muscovite Grade V1 was obtained from TED PELLA, INC (Redding, CA, USA).

### 2.2. Synthesis Procedure

**Figure 1:**
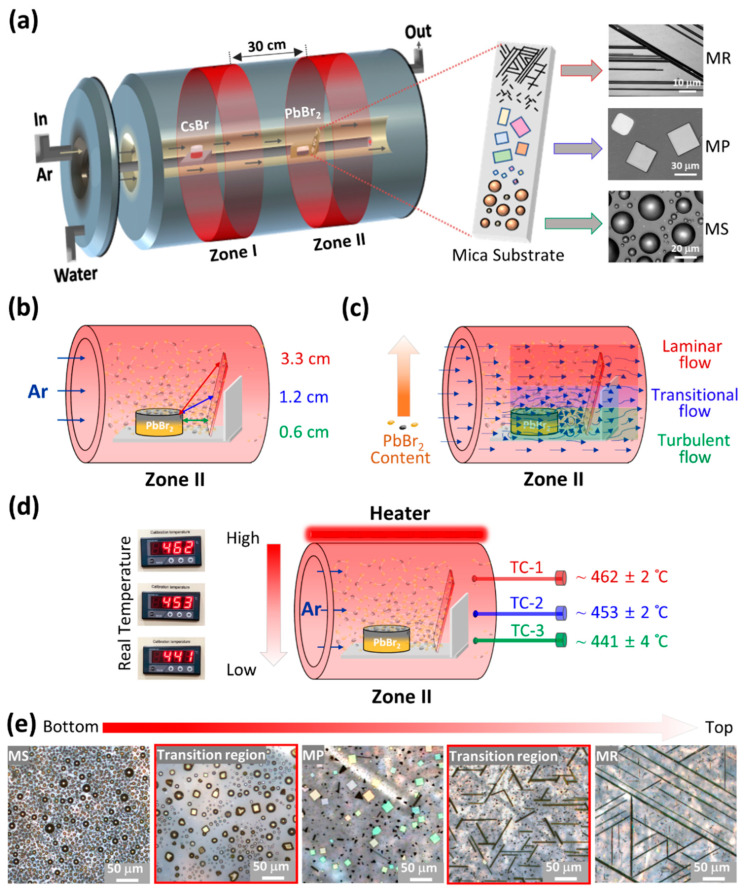
Illustration of the growth mechanism. (**a**) Schematic diagram of the two-temperature zone CVD furnace employed for the simultaneous yet selective growth of MR, MP, and MS across distinct regions of the vertically aligned mica substrate. (**b**) Schematic representation of zone II highlighting the variation in the distance between the PbBr_2_ crucible and different regions of the substrate. (**c**) Schematic illustration of zone II indicating the PbBr_2_ crucible-induced variation in the fluid flow dynamics and PbBr_2_ concentration along the length of the mica substrate. (**d**) Schematic illustration of the temperature gradient monitored via a set of three thermocouples at three distinct positions of the substrate. (**e**) A series of OM images scanned across the length of the substrate from bottom to top presenting a morphological transition from MS to MP to MR.

**Figure 2:**
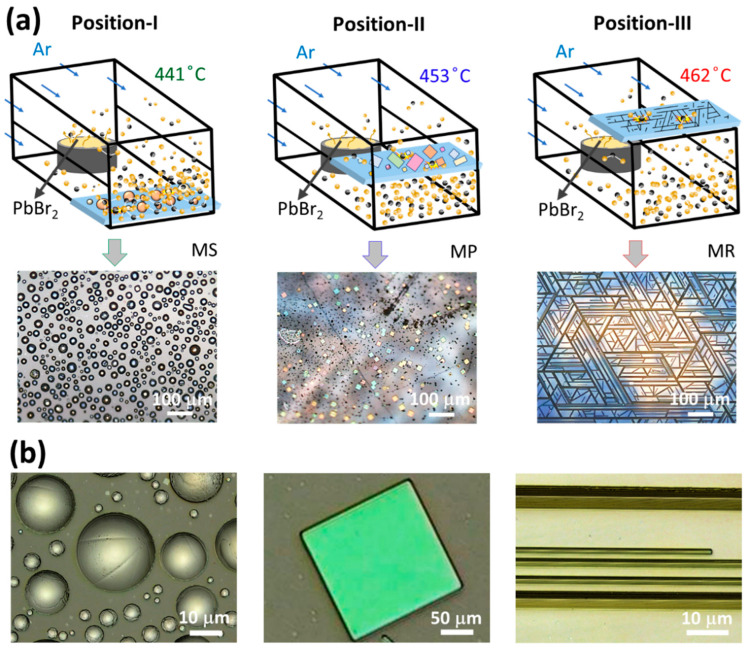
Cross-verification of selective growth. (**a**) Schematic illustration showing mica substrates placed horizontally at three discrete vertical positions mimicking the vertically aligned mica substrate of Figure 1. Note that the local conditions (temperature and precursor flux) at each of these positions are nearly uniform, but different with respect to each other. Consequently, a unique morphology is obtained entirely at each individual position with no mixing. For instance, MS is grown at position I, MP at position II, and MR at position III, as shown in the corresponding OM images. (**b**) Confocal microscope images of perovskite MS, MP, and MR geometry presenting sharp and featureless surfaces/edges signifying high crystallinity, while the relatively rough surface texture of MS geometry suggests low crystallinity.

CsPbBr_3_ microcrystals with various morphologies are grown via a two-zone, home-built CVD system as illustrated schematically in Figure 1a. Graphite crucibles containing CsBr and PbBr_2_ are placed in Zone I and II, respectively. A freshly cleaved (001) plane mica substrate is placed next to PbBr_2_ crucible in Zone II. Prior to deposition, the furnace is pumped down to a base pressure of ~10 mTorr, followed by argon (Ar-99.99%) gas purging to ensure the complete removal of air molecules/residues from the chamber. Subsequently, a working environment of ~760 Torr is established via a 150 sccm Ar flow rate. Once the working pressure is established, the temperature of the furnace is increased to the reaction temperature in two steps. Note that the reaction temperature for Zone I is relatively high, ~780 °C compared to Zone II ~465 °C, owing to the low volatility of CsBr [37]. In the first step, the temperature of Zone I and II is increased to 740 °C and 440 °C in 30 min, followed by an increase to 780 °C and 465 °C, respectively, in the next 10 min, as shown in Figure S1. Once the required reaction temperature is achieved, the Ar gas flow rate is reduced to ~40 sccm, while a constant reaction temperature is maintained for the next 20 min to realize the simultaneous growth of CsPbBr_3_ MS, MP, and MR at their selective regions across the mica substrate. Afterwards, the chamber is naturally cooled down to room temperature and samples are collected for further characterizations. Note that a series of experiments performed (Figure 2) to validate the selective growth followed the same recipe.

### 2.3. Characterizations

Energy dispersive X-ray spectroscopy (EDX) elemental mappings, scanning electron microscopy (SEM), transmission electron microscopy (TEM), HRTEM, high-angle annular dark-field scanning transmission electron microscopy (HAADF-STEM), and SAED analyses are conducted via an ARM200F microscope (JEOL BV, Nieuw-Vennep, The Netherlands). The specimens for the cross-sectional TEM analysis are prepared using a Helios NanoLab, conventional focused ion beam system (FIB, Thermo Fisher Sicientific Inc., Waltham, MA, USA) [38]. XRD patterns are obtained using a D8 Advance X-ray diffractometer manufactured by Bruker (Cu Kα radiation, λ  =  1.5406 Å), Karlsruhe, Germany. The room temperature steady-state photoluminescence (PL) spectra are acquired using a 473 nm continuous-wave laser as an excitation source, while the spectrum is analyzed by a DM500i monochromator equipped with three gratings (DongWoo Optron Inc., Gwangiu, Kyongi, Republic of Korea). The thickness profiles of the perovskite microcrystals are examined by a VK-X200, Keyence 3D laser scanning confocal microscope (Keyence Inc., Itasca, IL, USA). The energy density or fluence-dependent PL measurements are performed via a 355 nm passively Q-switched pico-second laser (PNV-M0250, Teem Photonics, Meylan, France) with a fixed pulse width of ~350 ps and a repetition rate of ~1 kHz. 

## 3. Results and Discussion

Figure 1a schematically illustrates the CVD method employed for the selective and simultaneous growth of CsPbBr_3_ microcrystals with various morphologies across different regions of the substrate. In contrast to conventional CVD approaches [25,31,34,35], where a single zone furnace is used to evaporate a mixture of CsBr and PbBr_2_, a two-zone CVD furnace is utilized to effectively vaporize the precursors owing to their distinct vapor pressures (Figure S2) [30]. The precursors (CsBr and PbBr_2_ powder) are placed in graphite crucibles 30 cm apart in high (zone I) and low (zone II) temperature zones, respectively. Argon (Ar) gas is used to transport CsBr vapors from zone I to II for the chemical reaction with PbBr_2_ vapors to form CsPbBr_3_, where a ~3.5 × ~1.25 cm^2^ mica substrate is aligned vertically next to the PbBr_2_ crucible (zone II) for nucleation. Interestingly, this configuration yields a varying precursor flux density and temperature along the substrate, offering regions suitable for the growth of different CsPbBr_3_ morphologies. This is because, unlike the reported CVD approaches, the precursor flux density here is not only controlled by changing the substrate-to-precursor distance but also by manipulating the fluid flow dynamics across different regions of the substrate, as illustrated in Figure 1b and Figure 1c, respectively. Owing to the relatively small separation between the PbBr_2_ crucible and the bottom of the substrate, the PbBr_2_ vapor concentration/flux density at the bottom region of the substrate is naturally higher and decreases gradually towards the top, as shown schematically in Figure 1c. This situation is further reinforced by the PbBr_2_ crucible-induced variation in the fluid flow dynamics [39,40], where the turbulent flow at the bottom region of the substrate transports a relatively high content of the precursors in comparison to the transitional and laminar flow at the center and top, respectively. Consequently, the higher flux densities do not offer sufficient time for the CsPbBr_3_ nuclei to follow substrate epitaxy, resulting in semi-sphere shaped microcrystals. On the other hand, the relatively moderate and lower precursor flux density at the center and top regions offer considerable time for the nuclei/seed to follow the substrate epitaxy, forming platelet and rod-shaped CsPbBr_3_ microcrystals, as shown schematically in Figure 1a. In addition to precursor flux density, the substrate temperature also plays a profound role in defining the geometry of the perovskite microcrystals. Figure 1d demonstrates the temperature gradient along the vertically aligned substrate monitored via a set of three thermocouples, while the temperature profile along the furnace length from zone I to II is shown in Figure S3. Contrary to the precursor flux density, the temperature decreases from top to bottom along the substrate, shown schematically in Figure 1d. This is because the top region of the mica substrate is closer to the heater and therefore exhibits a relatively high temperature (~462 ± 2 °C) compared to the middle/center (~453 ± 2 °C) and bottom (~441 ± 4 °C) regions. Generally, high temperatures favor MR growth over MP, while low temperatures and shorter substrate-to-precursor distances are suitable for MS growth [25,30,35]. Accordingly, MS, MP, and MR are grown at the bottom, center, and top regions of the substrate. Figure 1e presents a series of optical microscope (OM) images acquired along the length of the mica substrate from bottom to top, showing a clear transition from MS to MR geometry. The intermediate regions (highlighted by a red color) between these morphologies show irregularly shaped MS/MP and truncated MR, elucidating the flux density and temperature-dependent transition process. Moreover, Figure S4 shows that MR is distributed over a relatively large area (~1.5 cm) of the substrate owing to its lowest lattice mismatch compared to MP and MS. Even though MS has no epitaxy at all, it still covers the second largest area (~1.25 cm) on the substrate due to the high precursor flux at the bottom region of the substrate, as explained above. 

To further validate the role of precursor flux density and temperature in the selective growth of multiple CsPbBr_3_ geometries, a series of individual experiments is performed, where the mica substrates are horizontally placed at three discrete vertical positions referred to as position I: high flux density and low temperature (~441 °C), position II: moderate flux density and temperature (~453 °C), and position III: low flux and high temperature (~462 °C), as shown schematically in Figure 2a. As expected, MS, MP, and MR are grown on both facets of the mica substrate at position I, II, and III, respectively, as evidenced in their corresponding OM images, thus ensuring that the selective growth is consistent with the case of vertically aligned mica substrate (Figure 1). Furthermore, the topology of these perovskite geometries is examined via a 3D laser scanning confocal microscope. Figure 2b presents the confocal microscope images of MS, MP, and MR, respectively. These images readily reveal the triangular/square topology of MR/MP with sharp and well-defined edges demonstrating their high crystalline quality. On the contrary, MS presents a dome-shaped topography with a slightly rougher surface texture, indicative of a relatively low crystallinity owing to its non-epitaxial growth.

**Figure 3:**
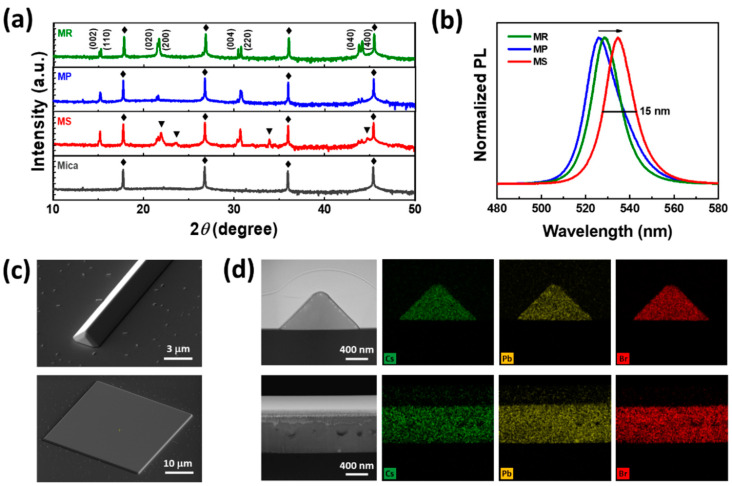
Structural and compositional analysis. (**a**) XRD pattern of MR, MP, and MS geometry grown on a mica substrate. The *θ*-2*θ* scans reveal that perovskite microcrystals crystallize into a pure orthorhombic phase with no secondary peaks of other structures. The additional peaks (indexed by delta) in the XRD pattern of MS geometry correspond to the residual or unreacted PbBr_2_ precursor. (**b**) Photoluminescence spectrum of MR, MP, and MS presents sharp peaks with a FWHM of ~15 nm, signifying excellent PL yield. (**c**) The bird’s-eye view SEM image demonstrates the sharp edge morphology and smooth surface texture of the as-grown CsPbBr_3_ MR and MP. (**d**) EDX elemental mappings of MR and MP confirm the presence of all the constituent elements, i.e., Cs (green), Pb (yellow), and Br (red).

Figure 3 presents the structural, optical, and compositional analyses of the as-grown perovskite microcrystals. Figure 3a displays the powder XRD patterns acquired from MS, MP, and MR dense regions of the mica substrate. Interestingly, all three geometries clearly present the four major diffraction peaks at 15°, 21°, 30°, and 44° with a characteristic peak-splitting representative of the orthorhombic phase of CsPbBr_3_ (ICSD 97851) [41,42]. However, additional peaks at 18.576°, 21.96°, 23.701°, and 33.932° (indexed by ▼) are observed only for MS geometry, indicating the presence of unreacted PbBr_2_, which is further validated by XRD (of each precursor), OM images, PL spectra, and EDX mapping shown in Figure S5, respectively. Note that the unreacted PbBr_2_ is formed mainly due to a high concertation of PbBr_2_ vapors at the bottom region of the substrate, as explained in Figure 1. Consequently, a handful of PbBr_2_ vapors nucleate before reacting with the incoming CsBr vapors, yielding silver-colored PbBr_2_ MS as shown in Figure S5. Moreover, the intense diffraction peaks of MR geometry at 21.721° and 44.162° (Figure S6) not only confirm the high density of MR (consistent with Figure S4) but also signify its epitaxial growth along the [001] direction of the mica substrate, as explained in the previous report on incommensurate epitaxial growth [30]. Figure 3b presents the PL spectra of the as-grown perovskite geometries. Clearly, all three geometries display a sharp PL peak at 535 nm (MS), 525 nm (MP), and 529 nm (MR), with a characteristic full width at half maximum (FWHM) ~15 nm, indicating the high PL quantum yield imperative for optoelectronic devices. Note that the slight redshift of the PL peak for MS geometry is attributed to its relatively high thickness, forcing the generated photons to reabsorb (photon recycling), a phenomenon widely observed in bulk perovskite crystals [43]. Figure S7 presents a detailed insight into the photon recycling of the individual geometries. Due to non-epitaxial growth and impurity peaks observed in the XRD, PL, and EDX mapping of MS, from this point forward, the focus of our study is limited to only MR and MP. In addition, Tauc’s plot as shown in Figure S12 indicating a bandgap of ~2.35 eV for the as-grown CsPbBr_3_ MP. Figure 3c presents the bird’s-eye view SEM image of the MR and MP geometry. These images reveal a smooth and featureless surface texture together with sharp edges/facets, highlighting their potential to form the self-assembled optical microcavities essential for the advanced photonic and optoelectronic applications, including strong exciton–photon coupling, polariton lasing, Bose–Einstein condensation, and many more [3]. The elemental composition of CsPbBr_3_ microcrystals is examined via EDX. Figure 3d presents the cross-sectional SEM images and the corresponding EDX mappings of MR and MP geometry. The mappings show a uniform distribution of the Cs (green), Pb (yellow), and Br (red) across the entire cross-section of the microcrystals. The relatively intense color mapping (red) suggests the high content of Br, consistent with the CsPbBr_3_ stoichiometry, analyzed quantitatively in Figure S8.

**Figure 4:**
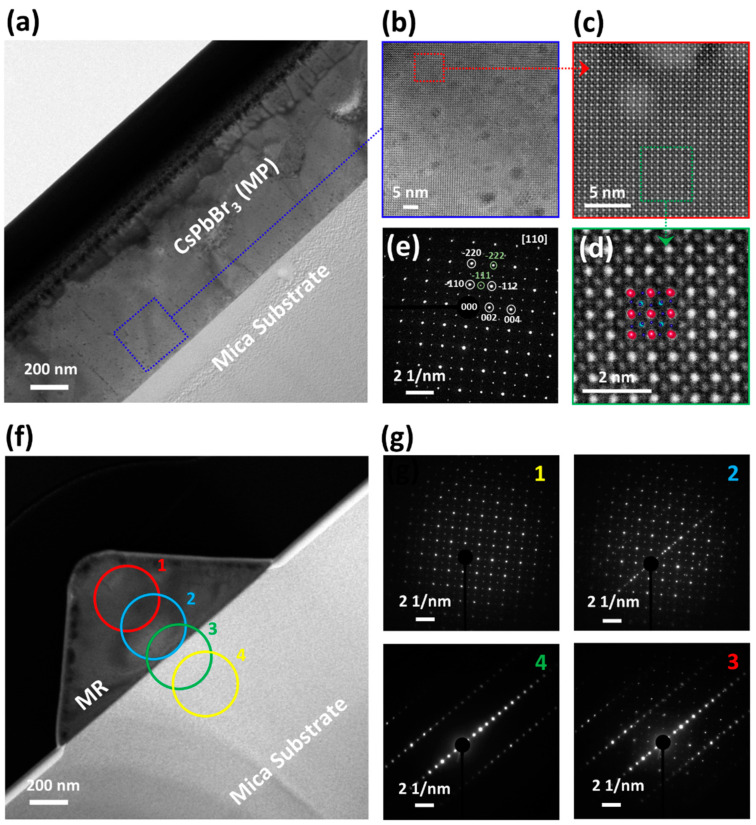
Structural and epitaxial growth analysis. (**a**) Low-resolution cross-sectional TEM image manifesting a sharp interface between the perovskite MP and mica. (**b**) High-resolution TEM image obtained from the highlighted area in (**a**). The image presents a uniform mesh texture with no clear sign of impurities/vacancies, confirming the purity of the as-grown perovskite MP. (**c**) The HAADF-STEM image acquired from the highlighted region in (**b**) further ensures the exquisite and defect-free atomic order of the perovskite MP (**d**) Magnified HAADF-STEM image from the highlighted region in (**c**) suggests that the atomic distribution of MP matches well with the orthorhombic phase of CsPbBr_3_ (ICSD-97851). (**e**) The SAED pattern demonstrates well-aligned spots without twinning and dislocation consistent with the lattice planes of the orthorhombic phase. (**f**) The cross-sectional TEM image of CsPbBr_3_ MR grown on mica substrate indicates a sharp and clean interface akin to MP geometry. (**g**) A series of SAED patterns (1–4) obtained by scanning the cross-sectional area from MR towards the mica substrate (marked in (**f**)) presents a transition from MR’s orthorhombic structure to mica’s layered structure, confirming the successful growth of the orthorhombic phase MR on the mica substrate. The inlet number of SAED pattern images refer to the corresponding cross-sectional TEM image.

To get a deeper insight into the crystal structure of the perovskite microcrystals at the atomic level, HRTEM was conducted. Figure 4a shows the cross-sectional TEM image of MP grown on the mica substrate, highlighting a sharp interface between the microcrystal and mica substrate. Figure 4b presents the HRTEM image obtained from the region highlighted (blue) in Figure 4a. The image shows a uniform mesh texture/pattern over the entire area ~ (50 × 50 nm^2^) with no clear sign of impurities/vacancies, confirming the high purity of perovskite MP. The HAADF-STEM image (Figure 4c) obtained from the highlighted region (red) of Figure 4b further verifies the exquisite and defect-free atomic order of the as-gown perovskite MP, wherein the magnified HAADF-STEM image (Figure 4d and Figure S9) suggests that the atomic distribution of MP geometry matches well with the orthorhombic crystal structure of CsPbBr_3_ [44]. Furthermore, the SAED pattern in Figure 4e demonstrates well-aligned diffraction spots without any twinning, dislocation, or stacking fault [45], confirming the orthorhombic phase of MP [41,42,46], consistent with HAADF-STEM and XRD analysis. Figure 4f presents the cross-sectional TEM image of CsPbBr_3_ MR grown on mica, indicating a sharp and clean interface akin to the MP geometry. Figure 4g presents a series of SAED patterns obtained by scanning the cross-sectional area from MR towards the mica substrate, as highlighted in Figure 4f (circle 1–4). These SAED patterns show a clear transition from MR’s orthorhombic structure to mica’s layered structure, confirming the successful growth of the orthorhombic phase MR on the mica substrate, consistent with the OM and XRD analysis.

**Figure 5:**
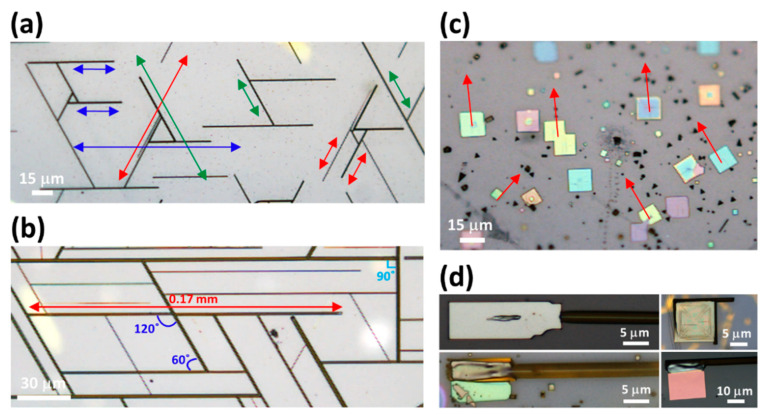
Detailed directional growth of MR and MP geometry following mica epitaxy. (**a**) OM image displaying the directional growth of MR on the mica substrate. MR prefer to grow along three specific orientations, forming equilateral triangles highlighted by red, blue, and green arrows. (**b**) Low-resolution OM images captured from another spot on the substrate, where MR exhibit lengths extending to a millimeter scale (0.17 mm) along preferred orientations, mostly forming 60° or 120° angles between the interconnected rods with occasional 90° intersections. (**c**) Low-resolution OM images indicating the directional growth of MP along specific orientations (red arrows) similar to MR. (**d**) Magnified OM images of coherently connected MR and MP sharing facets along the same growth direction further ensuring incommensurate epitaxial growth.

Figure 5a–d demonstrate the detailed directional growth of MR and MP geometry following the mica epitaxy. Figure 5a,b present the low-resolution OM images of MR grown on a mica substrate. It can be observed that MR prefer to grow along three specific directions (highlighted by red, blue, and green arrows) forming an equilateral triangle indicating 6-fold symmetry along the growth direction with the majority showing 60° or 120° angles between interconnected rods (Figure S10) [30]. Occasional 90° intersections are also noted in the MR network, which might be due to defects or the imperfect orientation of the atoms induced by stress or nucleation at the mica cleavage edge [31,47]. Likewise, the low-resolution OM image of MP geometry (Figure 5c) also demonstrates the growth along three preferred orientations (marked by red arrows) similar to MR. Occasionally, it is also observed that some MR and MP are coherently connected (Figure 5d), sharing the same facets, ensuring the incommensurate epitaxial growth owing to the low lattice mismatch between the orthorhombic phase CsPbBr_3_ and the mica substrate [30,31].

**Figure 6:**
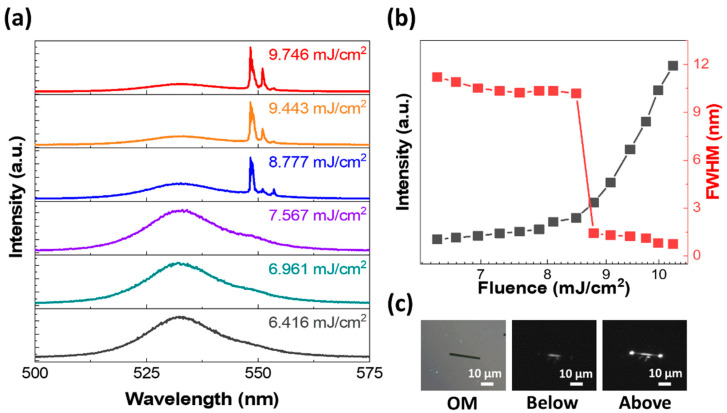
Lasing behavior in the MR geometry. (**a**) PL spectra with pump fluence increasing from 6.416 to 9.746 mJ/cm^2^. (**b**) Integrated intensity and FWHM are plotted as a function of pump fluence. The abrupt transition in both intensity and FWHM at a threshold value of ~8.77 mJ/cm^2^ confirms the lasing in MR geometry. (**c**) OM and the corresponding PL images of MR below and above the lasing threshold.

**Figure 7:**
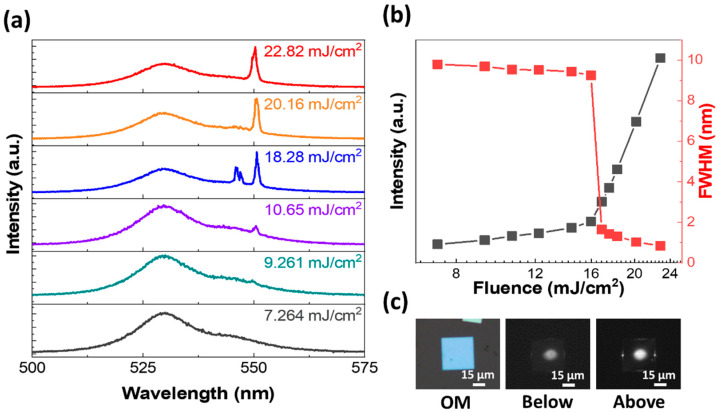
Lasing behavior in the MP geometry. (**a**) PL spectra with pump fluence increasing from 7.264 to 22.82 mJ/cm^2^. At a low pump fluence, a broad spontaneous emission peak dominates the spectrum. However, at high fluences, sharp peaks start to emerge that increase nonlinearly, signifying lasing actions. (**b**) Integrated intensity and FWHM are plotted as functions of pump fluence. The abrupt transition in both intensity and FWHM at a threshold value of ~16.8 mJ/cm^2^ confirms the lasing in the MP geometry. (**c**) OM and the corresponding PL images of MP below and above the lasing threshold.

Finally, to demonstrate the nonlinear optical characteristics of our as-grown perovskite microcrystals, we preform lasing measurements using a 355 nm pulsed laser (1 kHz, 350 ps). Figure 6a (Figure 7a) presents pump fluence dependent PL spectra of MR (MP) geometry. At a low pump fluence of ~6.416 (7.264) mJ/cm^2^, a broad spontaneous emission (SE) spectrum is observed with a FWHM of ~11 (9.7) nm. However, with an increase in the pump fluence, sharp peaks start to appear at the low energy edge of the SE band, dominating the spectrum nonlinearly, signaling conventional lasing actions in both MR and MP geometries. To ensure the photonic lasing action in our perovskite microcrystals, we plot integrated PL intensity and FWHM as a function of pump fluence, as shown in Figure 6b (Figure 7b). The abrupt change in PL intensity and the corresponding FWHM demonstrates a clear transition from SE to nonlinear lasing with threshold values of ~8.77(16.8) mJ/cm^2^. The relatively low lasing threshold of MR geometry is attributed to its high cavity quality factor (*Q*) ~730 compared to MP ~655. Figure 6c (Figure 7c) presents the OM and the corresponding PL images of MR (MP) above and below the lasing thresholds. The significant enhancement in the intensity of light emitting from the edges/ends above the threshold not only confirms the lasing behavior but also reveals information regarding the orientation of the optical microcavity. For instance, the two intense bright spots at the edges of the MP geometry suggest the formation of a Fabry–Perot (FP) cavity along the horizontal in-plane orientation. Likewise, the bright spots at the ends of MR geometry signify a waveguide type FP microcavity along the length of MR. Moreover, the size dependent lasing behavior for both MR and MP geometries is demonstrated in Figure S11. Thus, our work not only demonstrates a facile method for the simultaneous yet selective growth of multiple morphologies of CsPbBr_3_, but also validates their nonlinear optical characteristics, signifying the potential for practical device applications.

## 4. Conclusions

In summary, our study presents a comprehensive investigation into the controlled growth of CsPbBr_3_ microcrystals with diverse morphologies via a two-zone CVD system. Thanks to the unique configuration of our experimental setup, we are able to strategically tune the precursor flux density and temperature across the entire substrate by manipulating the substrate-to-precursor distance and the fluid flow dynamics. Consequently, microcrystals with different geometries (i.e., rod, platelet, and sphere) are simultaneously grown at the selective regions of the substrate, demonstrating the role of precursor flux density and temperature in defining the shape/geometry of the perovskite microcrystals. The high quality of our growth process is readily evident from confocal microscope and SEM images demonstrating a smooth surface texture along with well-defined facets/edges, particularly for MP and MR geometry. Furthermore, XRD, HR-TEM, and SAED patterns not only confirm the high crystallinity and orthorhombic phase, but also validate the epitaxial/directional growth of MR and MP geometry on a mica substrate. Finally, the nonlinear optical characteristics of the as-grown perovskite microcrystals are validated through low-threshold photonic lasing, demonstrating their potential for future optoelectronic applications.

## Data Availability

Data are contained within the article and Supplementary Materials.

## Supplementary Materials

The following supporting information can be downloaded at https://www.mdpi.com/article/10.3390/nano14151248/s1, Figure S1: Growth parameters employed in the CVD experimentation; Figure S2: Vapor pressure of the primary precursors (CsBr and PbBr_2_) with respect to temperature; Figure S3: Temperature profile of the CVD chamber along the furnace length; Figure S4: Vertically oriented mica substrate distribution with respect to various morphologies growth; Figure S5: Impurity identification in the MS through XRD, OM, PL and EDX mapping; Figure S6: XRD comparison of MR and MP grown on bare [001] mica; Figure S7: Thickness-dependent PL of CsPbBr_3_ MP, MR, and MS; Figure S8: EDX spectrum of CsPbBr_3_ MR and MP; Figure S9: Orthorhombic crystal lattice fitting over HAADF-STEM; Figure S10: OM image confirming the MR’s equilateral triangle formation and FFT image confirming its 6-fold symmetry; Figure S11: Effect of size-dependent lasing threshold for MR and MP; Figure S12: Tauc’s plot indicating a bandgap of ~2.35 eV for the as-grown CsPbBr_3_ microcrystals; and Table S1 presents a comparison chart of lasing threshold by considering various parameters. References [30,31,33,37,43,44,48,49,50,51,52,53,54,55,56,57,58] are cited in the Supplementary Materials.
