# Supplementary material for: Controlled Morphological Growth and Photonic Lasing in Cesium Lead Bromide Microcrystals
Source: Nanomaterials (Basel). 2024 Jul 25;14(15):1248. doi: 10.3390/nano14151248 (PMC11314365; doi:10.3390/nano14151248)
Supplement: Supplementary file 1 [file nanomaterials-14-01248-s001.zip › nanomaterials-3084078-supplementary-final version.pdf]

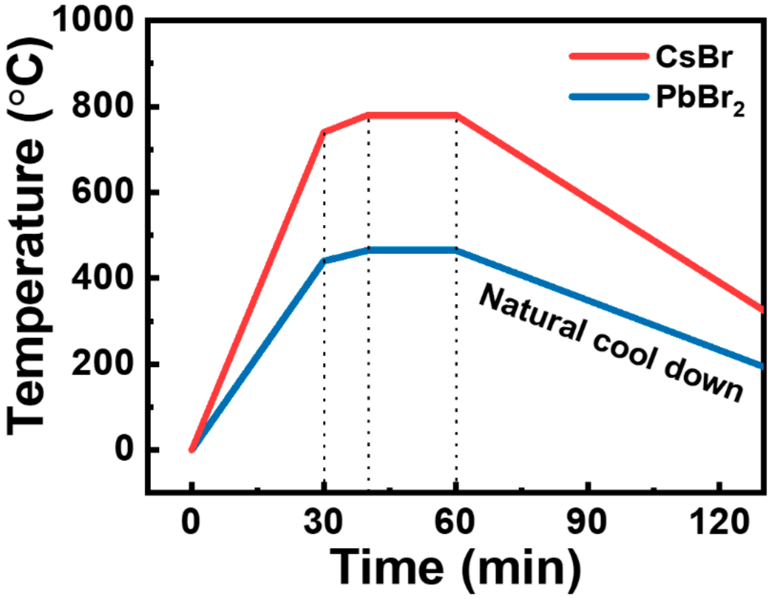


**Figure S1** illustrates the growth parameters employed in the chemical vapor deposition (CVD) experiments. Initially, both precursors CsBr and $PbBr_2$ are rapidly heated to the deposition temperature (CsBr at 740 °C and $PbBr_2$ at 440 °C) in an Ar environment over 30 minutes. Subsequently, a slower temperature increase is implemented to reach their respective nucleation temperatures (780 °C and 465 °C) over 10 minutes. Following successful deposition within 20 minutes, the CVD chamber is allowed to naturally cool down, resulting in the formation of the expected $CsPbBr_3$ in various morphologies.

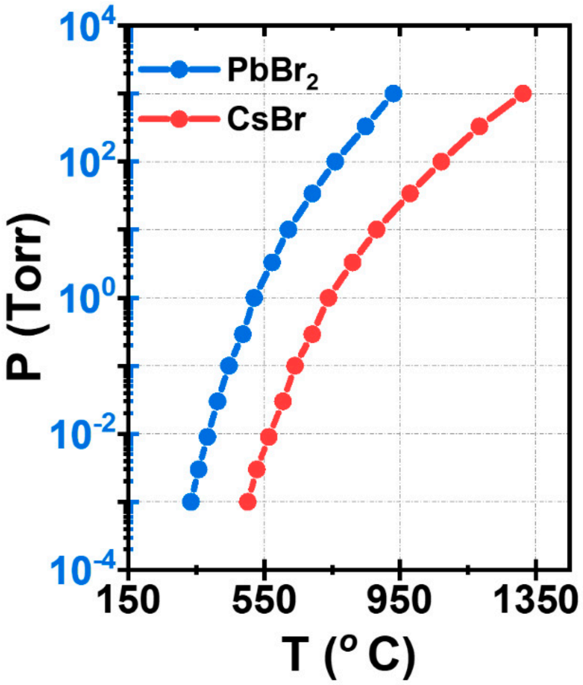


**Figure S2** illustrates the vapor pressure of the primary precursors (CsBr and $PbBr_2$) with respect to temperature [1]. In our CVD process (details in the experimental section), the CsBr precursor is subjected to a higher temperature regime (780 °C) compared to $PbBr_2$ (465 °C) to initiate the nucleation at the same time on the mica substrate. Note that the mica substrate is placed vertically close to the $PbBr_2$ precursor at a temperature varying region (440 °C–465 °C) as mentioned in Figure 1b.

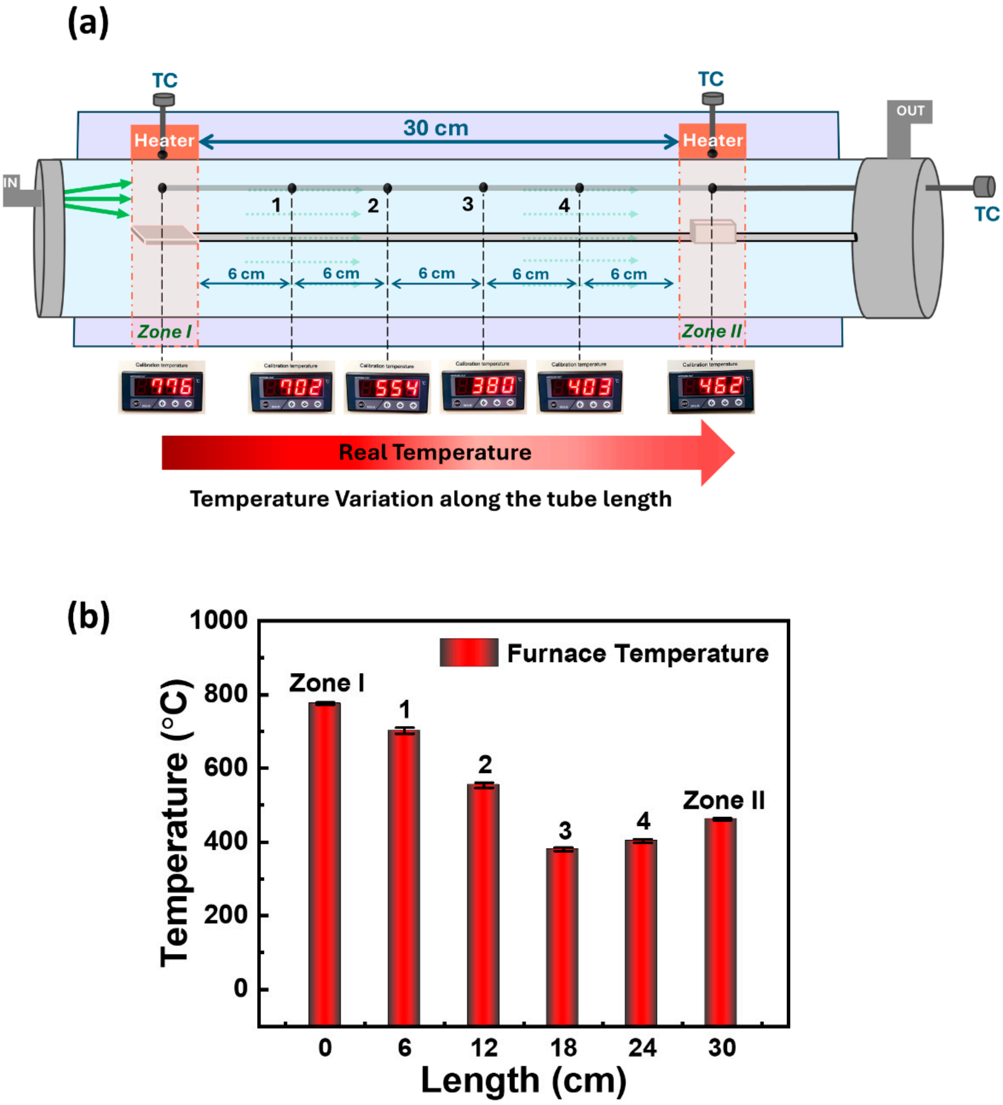


**Figure S3** illustrates the temperature profile of our CVD chamber along the furnace length. The temperature is monitored along the axis of the furnace at 6 different positions with an adjacent separation of ~6 cm. Figure S3(a) schematically presents the temperature gradient (real) along the axis of the furnace. As expected, a gradual decrease is observed as we move away from the source (Zone 1: 776 °C) towards the approximate center i.e. position 3: 380 °C. However, the temperature tends to rise again (position 4: 402 °C) as we approach the 2nd source (Zone II: 462 °C), as shown via the real temperature vs the furnace length plot in Figure S3(b). Note that this temperature

gradient has no significant effect on the growth dynamics, since the chemical reaction between the reactants and the subsequent growth process occur in Zone II where the temperature is relatively stable.

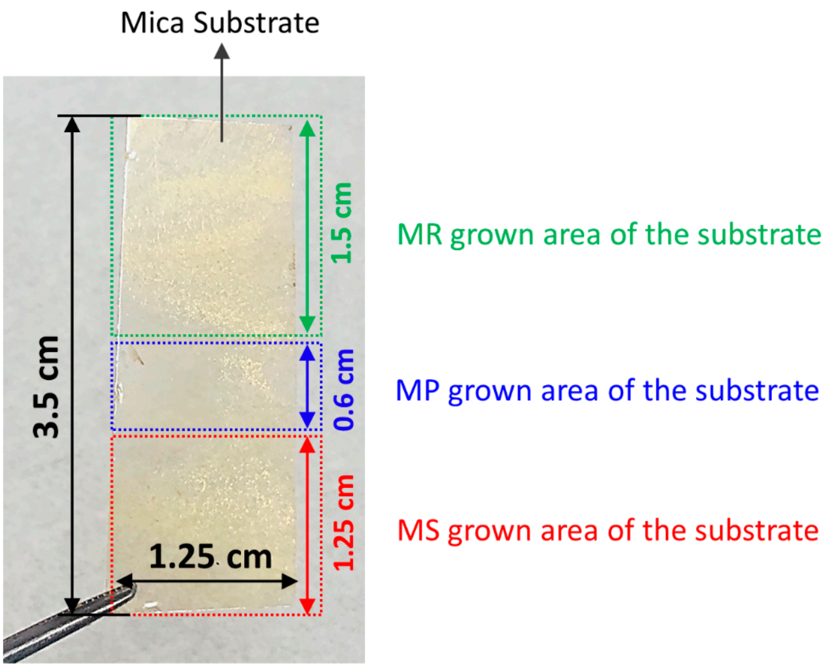


**Figure S4** shows the vertically oriented mica substrate distribution with respect to various morphological growth. The macroscopic observation suggests three distributed regions (green blue and red) corresponds to MR, MP, and MS growth areas, respectively. The MR extends over a major portion (1.5 cm) of the mica substrate compared to MSs (1.25 cm) and MPs (0.6 cm), indicating a favorable growth environment at the substrate's top region. This observation aligns with the MR dominant XRD peaks and directional growth following mica epitaxy.

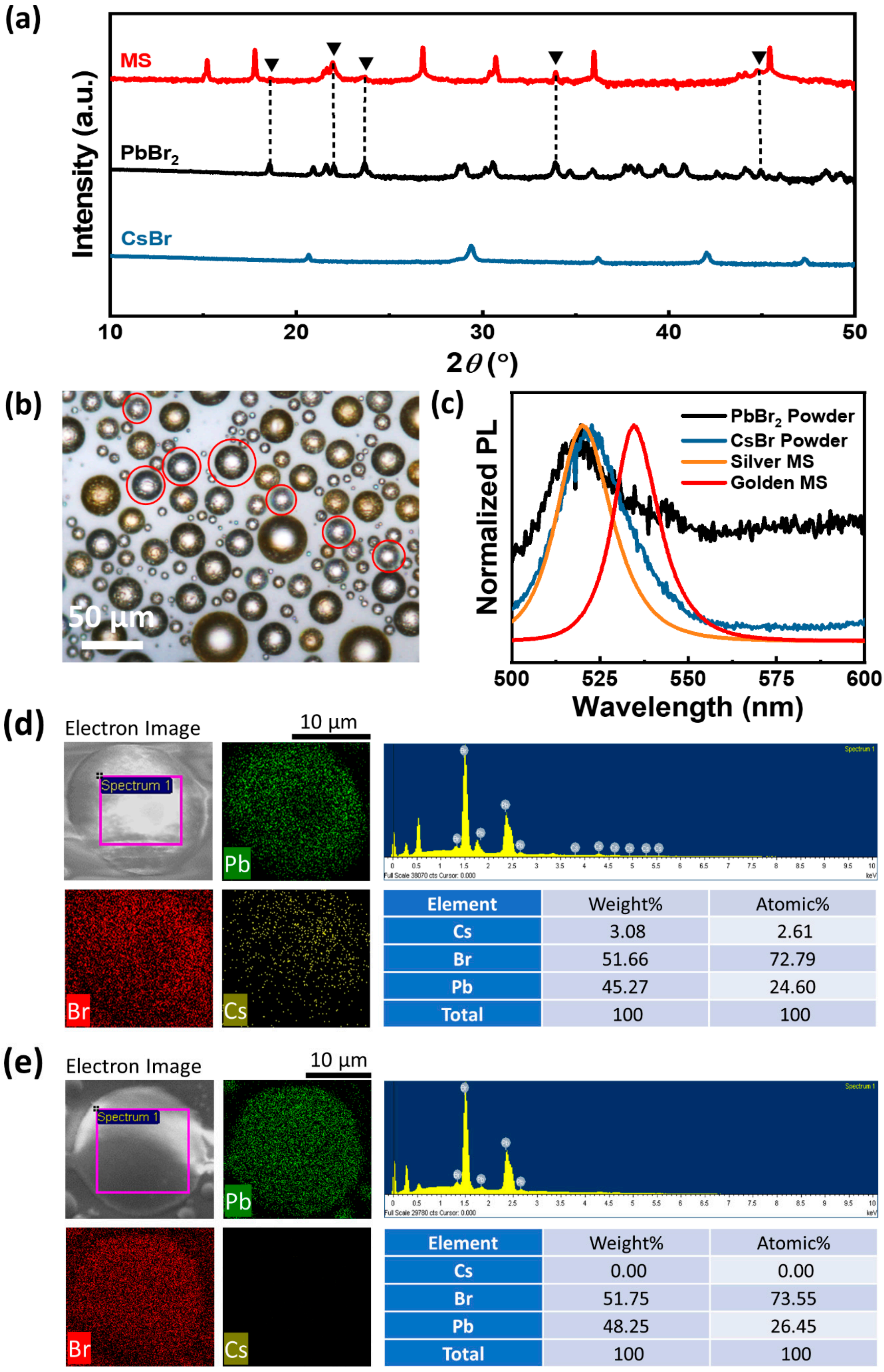


(d)

| Element | Weight% | Atomic% |
|---|---|---|
| Cs | 3.08 | 2.61 |
| Br | 51.66 | 72.79 |
| Pb | 45.27 | 24.60 |
| Total | 100 | 100 |

(e)

| Element | Weight% | Atomic% |
|---|---|---|
| Cs | 0.00 | 0.00 |
| Br | 51.75 | 73.55 |
| Pb | 48.25 | 26.45 |
| Total | 100 | 100 |

**Figure S5** illustrates impurity identification in the MS through XRD, OM, PL and EDX mapping. (a) XRD patterns compare θ-2θ scans of $CsPbBr_3$ MS with precursor powders (CsBr and $PbBr_2$) [2,3], revealing additional impurity peaks matching those of $PbBr_2$ powder, indicating direct nucleation of unreacted $PbBr_2$ during CVD. (b) The OM image from the bottom region of the mica substrate, where MS grow, shows silver and golden colors, suggesting the deposition of two distinct materials. (c) PL peaks from silver and golden MS, along with precursor powders, confirm that golden MS corresponds to $CsPbBr_3$, while silver MS aligns with $PbBr_2$ powder PL, consistent with XRD analysis. This confirms that the bottom region of the mica substrate contains not only $PbBr_2$ rich-MS but also unreacted $PbBr_2$-nucleated MS. Figure S5(d) and S5(e) present the elemental mappings and weight percentages of the golden and silver-colored MS, respectively. It is observed that golden MS exhibits a high content of Pb and Br compared to Cs, suggesting a $PbBr_2$ rich composition. On the other hand, silver MS shows no sign of Cs at all and the existence of only Pb and Br elements confirms its $PbBr_2$ composition. The formation of unreacted $PbBr_2$ MS (silver) is indirect evidence of a high content/flux density of $PbBr_2$ vapors at the bottom of the substrate. Consequently, plenty of $PbBr_2$ vapors do not get sufficient time to react with incoming CsBr vapors and nucleate before reaction, resulting in the growth of unreacted $PbBr_2$ MS (silver color). Unlike MS, the relatively lower content of $PbBr_2$ vapors at the center and top regions of the substrate offer favorable conditions for the stoichiometric growth of MP and MR geometry with no excess or deficiency of $PbBr_2$ (Figure S8).

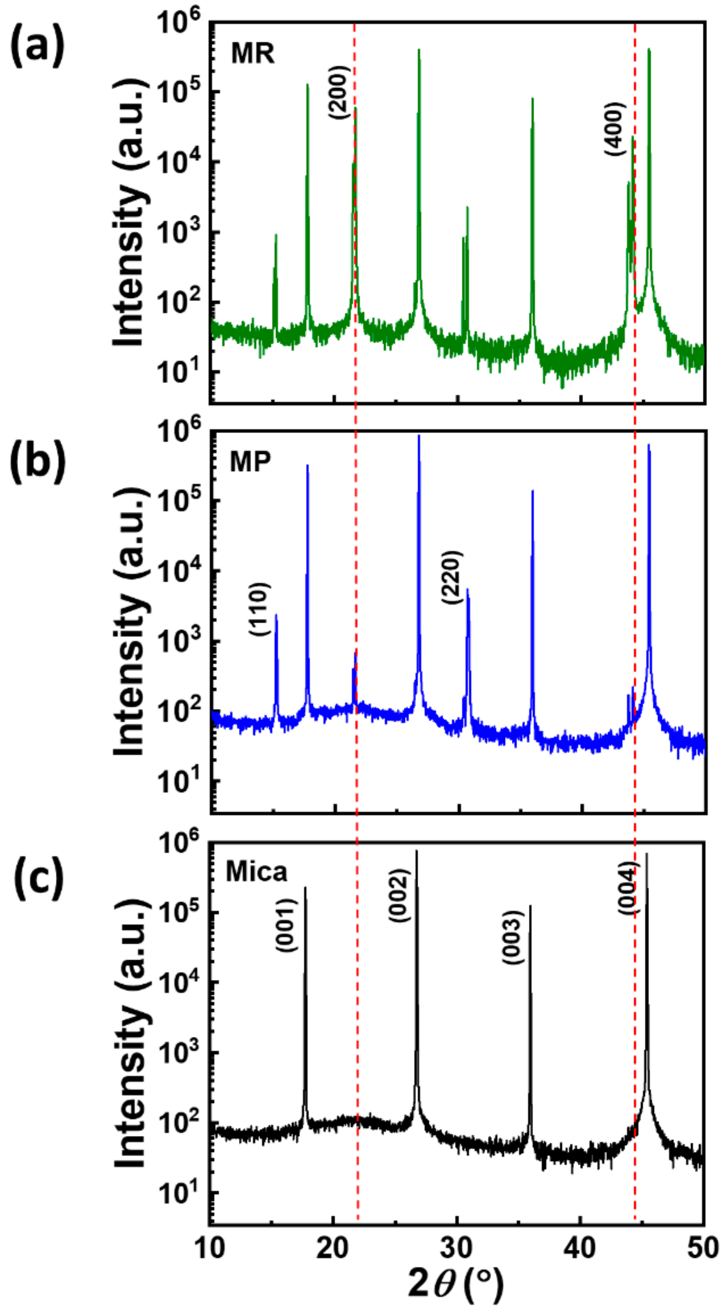


**Figure S6** presents the XRD comparison of MR and MP grown on bare [001] mica. (a-c) Through the comparative analysis of XRD peak intensities with morphological growth, we identify that the strong (200) and (400) peaks (highlighted with a red dashed line) come from large-scale MR arrays and the less intense peak from the less prevalent MP, resulting in a very interesting observation that when the growth switches from 2D MP

to 1D MR, the out of-plane direction alters from (110) to (200) [4]. This observation is consistent with Figure S4, which shows the substrate to morphology growth ratio of MR is larger while MP is lower.

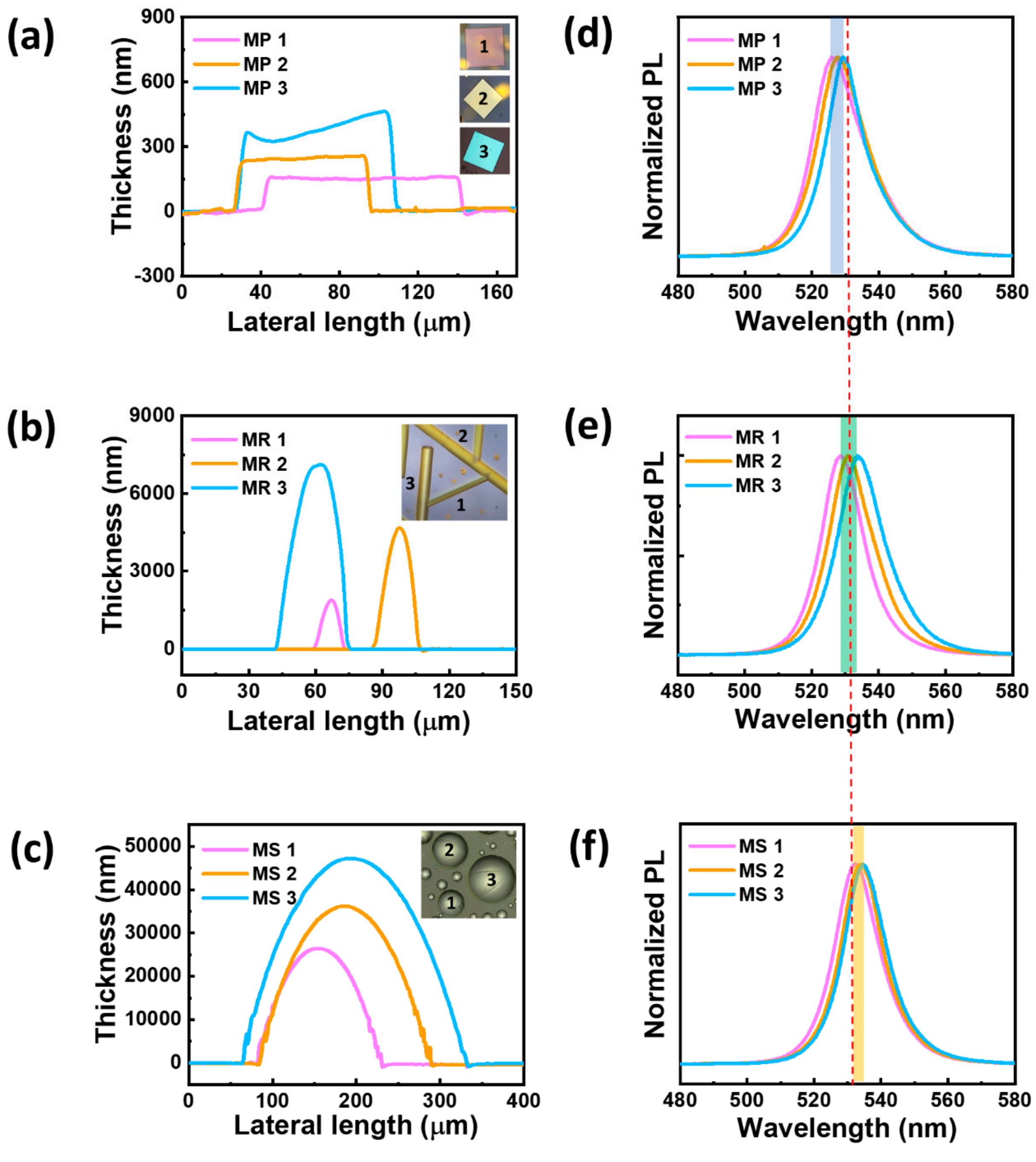

**Figure S7** sequentially illustrates the thickness-dependent photoluminescence (PL) of $CsPbBr_3$ MP, MR, and MS. Panels (a-c) depict the thickness of the three morphologies, as shown in the inset optical microscopy (OM) images. MP exhibits thicknesses ranging from 100 to 400 nm, indicating 2D growth compared to the thicker MR and MS in the micrometer range. Panels (d-f) show the corresponding PL spectra for each morphology, indicating a slight red shift with increasing thickness due to PL reabsorption in bulk crystals, a phenomenon known as photon recycling [5].

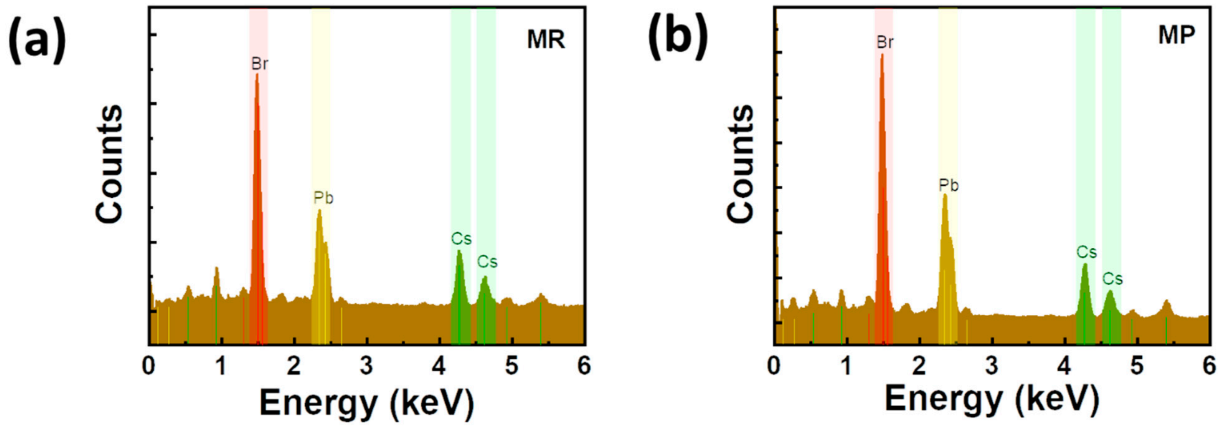


| Element | Weight% | Atomic% |
|---|---|---|
| Cs | 26.31 | 22.14 |
| Pb | 29.83 | 16.15 |
| Br | 43.86 | 61.71 |
| Total | 100 | 100 |

| Element | Weight% | Atomic% |
|---|---|---|
| Cs | 26.24 | 21.98 |
| Pb | 29.84 | 16.23 |
| Br | 43.92 | 61.79 |
| Total | 100 | 100 |

**Figure S8** shows the EDX spectrum of $CsPbBr_3$ (a) MR and (b) MP indicating the relative content ratio of the as grown samples.

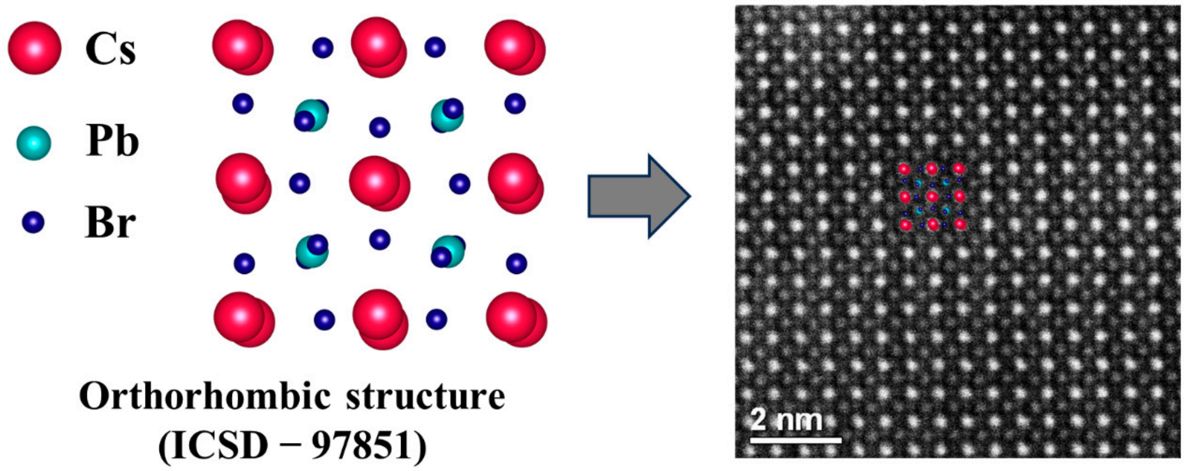


**Figure S9** showcases high-angle annular dark-field scanning transmission electron microscopy (HAADF-STEM), revealing a defect-free crystal structure in MP. The schematic atomic arrangement of the orthorhombic $CsPbBr_3$ crystal structure, acquired via Vesta software, perfectly aligns with the atomic positions observed in the HAADF-STEM image [6]. The FFT image in the inset indicates the 6-fold symmetry of the as-grown MR on the mica substrate.

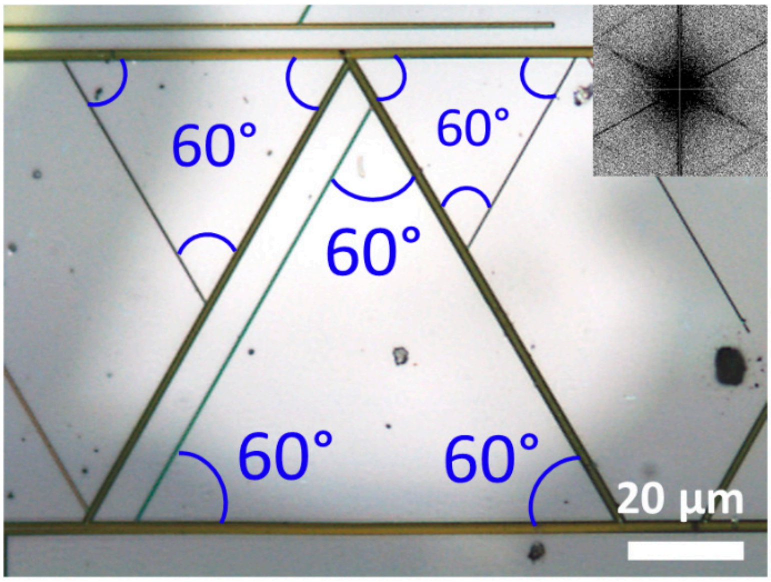

**Figure S10** depicts an optical microscopy (OM) image confirming the equilateral triangle formations within the network of MR, following distinct orientations along three preferred directions on the mica substrate [7].

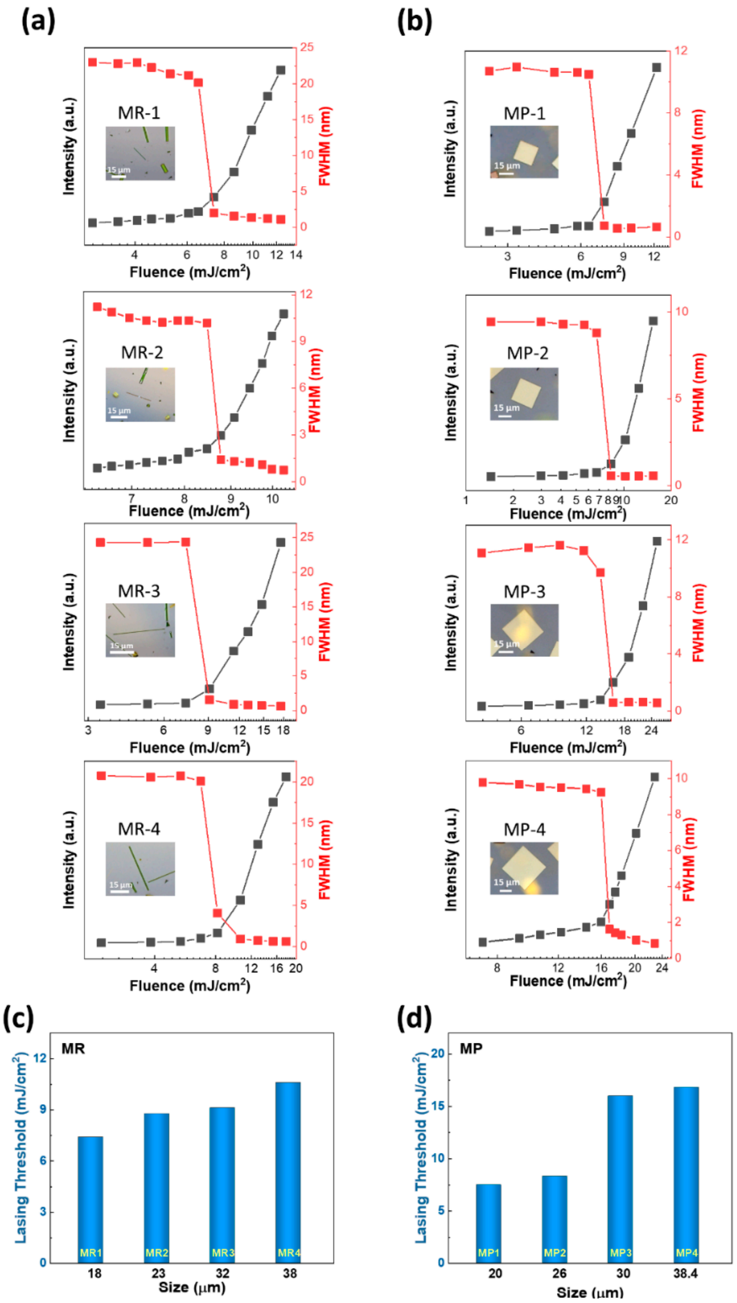


**Figure S11** depicts the effect of a size-dependent lasing threshold for MR and MP. Cavities constitute an integral part of the laser. In general, cavities are characterized by

two main quantities: the modal volume V, and the quality factor Q. In many applications, high Q's and small V's are highly desirable to achieve high finesse essential for low threshold lasing and high Purcell factor. The quality factor Q is independent of the cavity size, while the modal volume directly correlated to the physical dimensions of the cavity. Cavities with smaller physical dimensions yield a low V and vice versa [8,9,10]. Therefore, to investigate the size effect, lasing behavior in MR and MP with different sizes are measured as shown in (a) and (b) respectively. Clearly, MR(c) and MP(d) with smaller physical dimensions present a low lasing threshold compared to their larger counterparts, signifying the role of microcrystal size on lasing behavior, consistent with the literature.

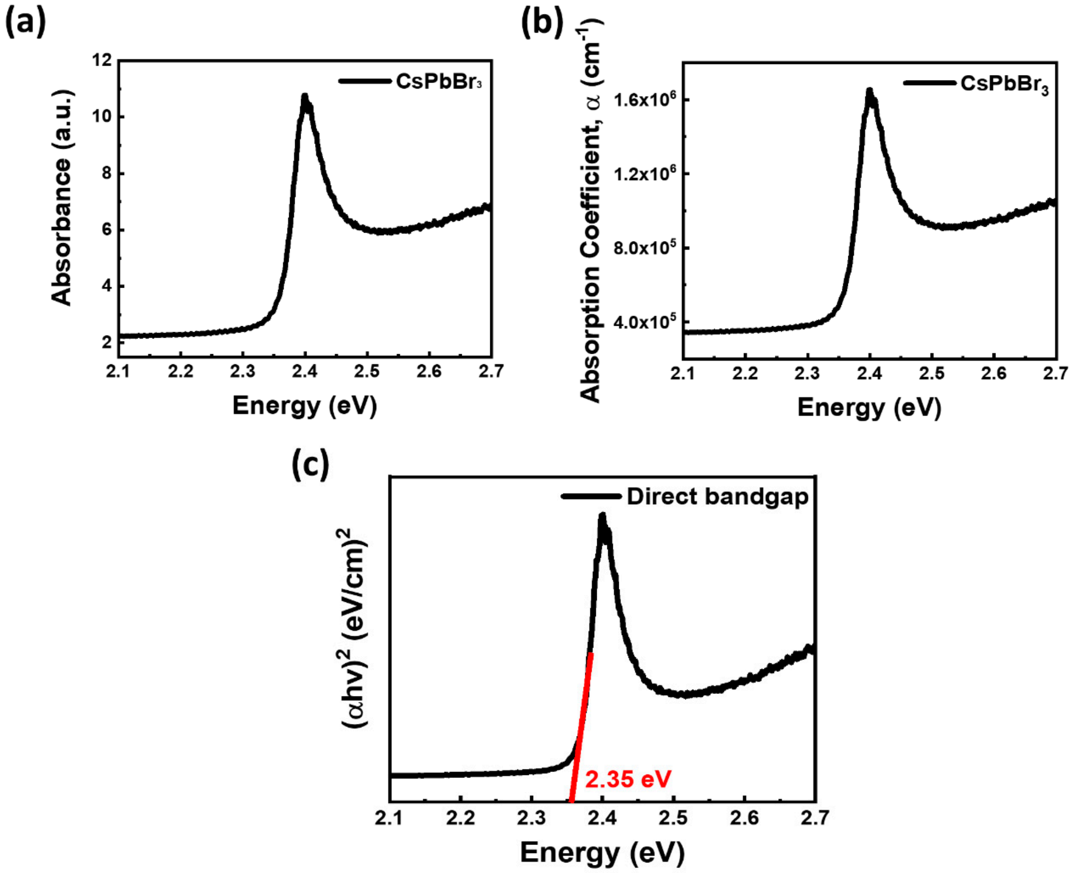

**Figure S12:** Tauc's plot indicating a bandgap ~2.35 eV for the as-grown $CsPbBr_3$ microcrystals. (a) The absorbance vs energy plot represents the absorption spectrum of $CsPbBr_3$. (b) The absorption coefficient vs energy plot extracted from (a) gives absorption coefficient values across the entire spectrum (c) Tauc's plot indicating a bandgap of ~2.35 eV calculated from (a) and (b).

**Table S1** displays a comparative analysis of the lasing threshold across various geometries, namely MR, MP, MS, and thin film, while considering numerous synthesis processes (growth method).

| No. | Growth Method | Morphology | Lasing Threshold | Reference |
|---|---|---|---|---|
| 1 | Solution-based synthesis | Nanowires | ~63.86 μJ $cm^{-2}$ | [11] |
| 2 | Inkjet-printing synthesis | Microplates | ~4.0 μJ $cm^{-2}$ | [12] |
| 3 | Chemical vapor deposition (CVD) | Microsphere | ~3.5 μJ $cm^{-2}$ | [13] |
| 4 | Solution-based synthesis | Nanocrystals | ~254 μJ $cm^{-2}$ | [14] |
| 5 | CVD | Hemisphere | ~36.1 μJ $cm^{-2}$ | [15] |
| 6 | Solution-based synthesis | Thin film | ~450 μJ $cm^{-2}$ | [16] |
| 7 | CVD | Microplates | ~93 μJ $cm^{-2}$ | [17] |
| **8** | **CVD** | **Microplate (MP)**<br>**Microrod (MR)** | **7.48 mJ $cm^{-2}$**<br>**7.38 mJ $cm^{-2}$** | **This Work** |

References:


1. LUXEL Vapor Pressure Chart. Available online: http://luxel.com/wp-content/uploads/2013/04/Luxel-Vapor-Pressure-Chart.pdf (accessed 26 December 2023).
2. Torres, O.; Gordillo, G.; Plazas, M.; Landínez Téllez, D.; Roa-Rojas, J. Optical features of $PbBr_2$ semiconductor thin films for radiation attenuation application. *J Mater Sci. Mater. Electron.* **2021,** *32,*16937-16944.
3. Ravikant, C.; Arun, P.; Kumar, K. SPR sensitivity of silver nanorods in CsBr-Ag nanocomposite thin films. *Mater. Res. Express* **2016,** *3*, 076403.
4. Wang, Y.; Sun, X.; Shivanna, R.; Yang, Y.; Chen, Z.; Guo, Y.; Wang, G. C.; Wertz, E.; Deschler, F.; Cai, Z.; Zhou, H.; Lu, T.M.; Shi, J. Photon transport in one-dimensional incommensurately epitaxial $CsPbX_3$ arrays. *Nano Lett.* **2016**, *16*, 7974-7981.
5. Yamada, T.; Yamada, Y.; Kanemitsu, Y. Photon recycling in perovskite $CH_3NH_3PbX_3$ (X= I, Br, Cl) bulk single crystals and polycrystalline films. *J. Lumin.* **2020,** *220*, 116987.
6. Brennan, M.C.; Kuno, M.; Rouvimov, S. Crystal structure of individual $CsPbBr_3$ perovskite nanocubes. *Inorg. Chem.* **2018**, *58*, 1555-1560.
7. Chen, J.; Fu, Y.; Samad, L.; Dang, L.; Zhao, Y.; Shen, S.; Guo, L.; Jin, S. Vapor-phase epitaxial growth of aligned nanowire networks of cesium lead halide perovskites ($CsPbX_3$, X= Cl, Br, I). *Nano Lett.* **2017**, *17*, 460-466.
8. Lalanne, P.; Mias, S.; Hugonin, J.P. Two physical mechanisms for boosting the quality factor to cavity volume ratio of photonic crystal microcavities. *Opt. Express* **2004**, *12*, 458-467.

9. Khurgin, J.B.; Noginov, M.A. How Do the Purcell Factor, the Q-Factor, and the Beta Factor Affect the Laser Threshold? *Laser & Photonics Rev.* **2021**, *15*, 2000250.

10. Srinivasan, K.; Borselli, M.; Painter, O.; Stintz, A.; Krishna, S. Cavity Q, mode volume, and lasing threshold in small diameter AlGaAs microdisks with embedded quantum dots. *Opt. Express* **2006**, *14*, 1094-1105.

11. Li, Y.; Guan, S.; Liu, Y.; Xu, G.; Cai, B. Lasing properties of cesium lead halide perovskite nanowires fabricated by one-drop self-assembly and ion-exchange methods. *Opt. Express* **2018**, *26*, 33856-33864.

12. Gu, Z.; Zhou, Z.; Huang, Z.; Wang, K.; Cai, Z.; Hu, X.; Li, L.; Li, M.; Zhao, Y.S.; Song, Y. Controllable growth of high-quality inorganic perovskite microplate arrays for functional optoelectronics. *Adv. Mater.* **2020**, *32*, 1908006.

13. Du, W.; Zhang, S.; Wu, Z.; Shang, Q.; Mi, Y.; Chen, J.; Qin, C.; Qiu, X.; Zhang, Q.; Liu, X. Unveiling lasing mechanism in $CsPbBr_3$ microsphere cavities. *Nanoscale* **2019**, *11*, 3145-3153.

14. Yang, L.; Wang, T.; Min, Q.; Pi, C.; Li, F.; Yang, X.; Xu, X. Ultrahigh photo-stable all-inorganic perovskite nanocrystals and their robust random lasing. *Nanoscale Adv.* **2020**, *2*, 888-895.

15. Zhang, H.; Zhao, C.; Chen, S.; Tian, J.; Yan, J.; Weng, G.; Hu, X.; Tao, J.; Chen, S.; Akiyama, H.; Chu, J. Lasing operation in the $CsPbBr_3$ perovskite micron hemisphere cavity grown by chemical vapor deposition. *Chem Eng J.* **2020**, *389*, 124395.

16. Yakunin, S.; Protesescu, L.; Krieg, F.; Bodnarchuk, M.I.; Nedelcu, G.; Humer, M.; Luca, G.D.; Fiebig, M.; Heis, W.; Kovalenko, M.V. Low-threshold amplified spontaneous emission and lasing from colloidal nanocrystals of caesium lead halide perovskites. *Nat. commun.* **2015**, *6*, 8056.

17. Wang, J.; Yu, H.; Liu, G.; Liu, W.; Duan, Y.; Cheng, X.; Dai, L.; Wang, S.; Gong, Q. Ultrafast lasing dynamics in a $CsPbBr_3$ perovskite microplate. *Adv. Photonics Res.* **2022**, *3*, 2100182.
